# Patients’ appreciation of pre-implant augmentation of the severely resorbed maxilla with calvarial or anterior iliac crest bone:a randomized controlled trial

**DOI:** 10.1186/s40729-019-0185-3

**Published:** 2019-09-30

**Authors:** Dagmar E. Wortmann, Carina G. Boven, Jurjen Schortinghuis, Arjan Vissink, Gerry M. Raghoebar

**Affiliations:** 1 Department of Oral and Maxillofacial Surgery, University of Groningen, University Medical Centre Groningen, PO Box 30.001, 9700 RB Groningen, The Netherlands; 2 Department of Oral and Maxillofacial Surgery, Treant Scheper Ziekenhuis Emmen, Emmen, The Netherlands

**Keywords:** Patient satisfaction, PROM, Autogenous bone graft, Edentulous atrophic maxilla, RCT, Iliac crest, Calvarium

## Abstract

**Background:**

Little is known about the impact of bone graft harvesting for pre-implant augmentation of the maxilla from a patient’s perspective. To assess patient-reported outcome measures (PROMs) related to augmentation of the extremely resorbed edentulous maxilla with calvarial or anterior iliac crest bone.

**Materials and methods:**

For this randomised controlled trial, 20 consecutive edentulous patients needing extensive pre-implant surgery of the maxilla were randomly assigned to either calvarial (*n* = 10) or anterior iliac crest (*n* = 10) bone harvesting. Patient reports on procedure-related satisfaction, questionnaires on oral functionality (denture satisfaction, chewing ability) and oral health-related quality of life (OHIP-49NL) and subjective donor site-related outcomes (e.g. of post-operative pain, scar formation, physical mobility) were assessed.

**Results:**

Irrespective of the harvesting site, patients were generally satisfied (median VAS score 93 (86–99) mm, *p* = 0.400) with the procedure and its final results. Post-operative pain was mild (median 40 (20–40) mm) and decreased to no pain (4 (0–16) mm) within 14 days. Early post-operative pain was significantly higher following anterior iliac crest harvesting (*p* < 0.00). Impact on physical mobility, daily functioning and satisfaction with the scar formation were similar in both groups.

**Conclusions:**

The assessed PROMs confirmed that bone graft harvesting from the calvarium or anterior iliac crest is an appropriate procedure, reflected by high levels of satisfaction, minor long-term sequela and improvement of perceived oral health. For clinical decision-making, decisions can be based on individual features and preferences.

**Trial registration:**

NTR, NTR3968, registered 1 July 2013.

## Background

Pre-implant augmentation of the maxilla using extraorally grafted bone has been studied objectively on medical indicators, such as surgical complication rate, donor site morbidity and physical characteristics [1–4]. Little is known about the patients’ perceptions of the applied bone harvesting techniques for reconstruction of the maxilla [5]. Moreover, the studies performed thus far assessing patients’ perspectives have been mainly retrospective [6–10].

The use of objective outcome measures strikes to the modern view on clinical research that appropriate judgments on the outcome of therapeutic procedures come from those who experience them from beginning to end, i.e. the patients themselves [11]. Hence, the use of patient-reported outcome measures (PROMs) to assess patients’ opinion on healthcare has been set as a standard in treatment evaluation. As a result, patient satisfaction ratings have become important indicators for therapeutic efficacy [12].

PROMs have shown that an edentate state is associated with a significant decrease in oral health-related quality of life (OHRQoL) [13, 14] and that adequate prosthetic treatment results in improvement in OHRQoL and patients’ satisfaction [5, 13, 14]. The introduction of implant-supported overdentures has been a great asset in resolving problems related to a maxillary denture [13–15]. Implant placement in the extremely resorbed maxilla usually requires, however, augmentation with extraorally grafted bone. While there is ample evidence that the PROMs are favourable regarding replacement of a conventional maxillary denture with a maxillary overdenture on implants, scarce information is available in terms of how patients experience the bone harvesting procedure enabling maxillary augmentation surgery. Therefore, the aim of this prospective study was to assess patient satisfaction and patient-reported morbidity of patients needing calvarial or anterior iliac crest bone harvesting to reconstruct an extremely resorbed edentulous maxilla before being treated with an implant-retained maxillary overdenture.

## Methods

### Patient population

A total of 20 consecutive eligible patients was asked to join the study. These patients were referred to the Department of Oral and Maxillofacial Surgery of the University Medical Centre Groningen (UMCG), Groningen, the Netherlands, having problems with wearing an upper denture (pain, mobility, loss of retention). These problems were a result of severe resorption of the edentulous maxilla. Patients were included when insufficient bone volume was available for reliable implant placement, that is, < 3 mm bone height in the maxillary sinus area and < 2 mm bone width in the anterior maxillary area. The bone height and width were assessed using cone-beam computed tomography (CBCT) scanning. For the temporal bone, the thickness in the area between the articular tubercle and the end of the mastoid bone had to be > 5 mm to allow for harvesting calvarial bone. None of the participants had undergone an operation at the donor site before.

### Design of the study

Twenty patients gave written consent to participate in the study. Randomisation software was applied to divide them into two groups based on the location for harvesting the bone grafts: the calvarium (*n* = 10) or the anterior iliac crest (*n* = 10). All patients were subjected to a bilateral maxillary sinus floor augmentation and reconstruction of the width of the maxilla. The surgeries took place between November 2014 and September 2016. Each patient was followed up until at least 12 months.

**Fig. 1:**
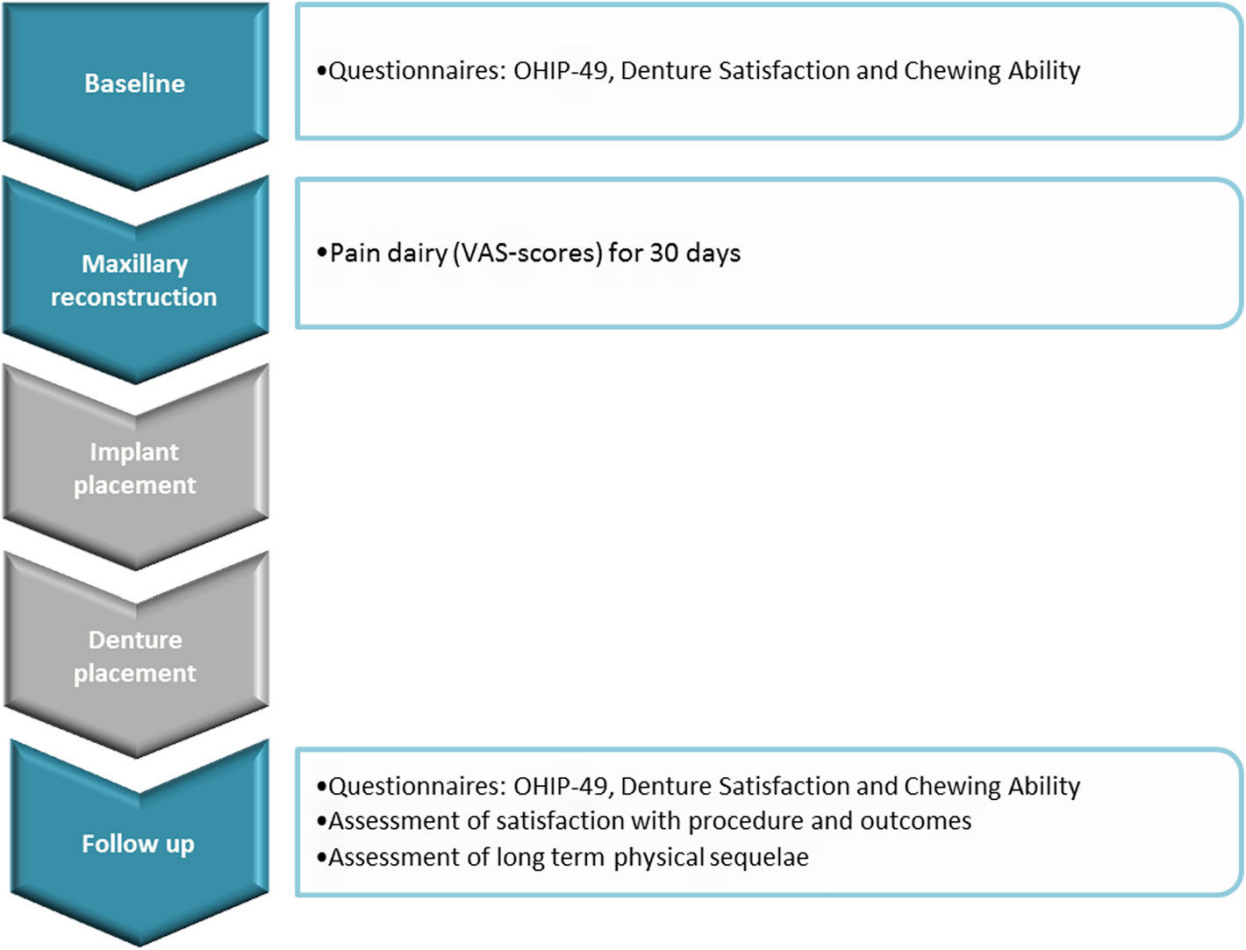
Timing of PROM assessments in treatment programme. The PROMS were assessed at pre-defined steps in the treatment programme of an individual participant. First, when a participant was included for the study but before an intervention had taken place, the OHIP-49NL, denture satisfaction and chewing ability questionnaires were administered to determine the baseline level of oral health-related quality of life, satisfaction with the current denture and perceived ability to chew food, respectively. Next, directly following the reconstruction surgery that included the bone graft harvesting from either calvarium or anterior iliac crest, the post-operative pain was assessed by asking participants to report the perceived pain at donor site on a 100-mm VAS score for 30 days. Following a 4-month osseointegration phase, the implants were placed in the reconstructed maxilla. Another 4 months later, the patients received their implant-retained denture. No PROMs were assessed during these two steps as they were not related to the bone graft harvesting surgery. Finally, at a 12-month follow-up meeting, the OHIP-49NL, denture satisfaction and chewing ability questionnaires were administered again to measure improvement or decrease in scores. Moreover, patient satisfaction with the procedure and the outcomes were assessed as well as presence of long-term physical sequelae resulting from the bone graft harvesting procedure

PROMs were assessed at several moments in time (Fig. 1). To control for equality between groups and determine improvement in perceived oral health, OHRQoL, denture satisfaction and chewing ability were assessed at baseline and 12 months post-treatment. Furthermore, post-operative pain was assessed during the first 30 post-operative days. At the 12-month follow-up meeting, patient-reported satisfaction and donor site-related outcomes were assessed too.

The study protocol was reviewed and approved by the medical ethical committee of the UMCG, reference NL48614.042.14. Written consent was obtained from all participants.

### Surgical procedures

To harvest calvarial bone, a full-thickness flap was raised, followed by marking the outer table graft with a burr until the diploe was encountered. With a bone scraper (SafeScraper Twist; META, Reggio Emilia, Italy), a bevel was created through around the calvarial outer table graft to facilitate its removal with a reciprocating saw. The scraper was used to collect copious amounts of cancellous-like bone. To remove the graft without breaking, parallel saw cuts were made in situ [16]. Next, the graft was removed piece by piece. The ensuing defect in the skull was reconstructed with bone cement (Palacos®, Zimmer Biomet, Warsaw, IN, USA). For harvesting anterior iliac crest bone, an incision was made from 1 cm behind the anterior superior iliac spine toward posteriorly, following the iliac crest. It was continued sharply to the midcrest, separating the aponeurosis of the fascia lata and the oblique abdominal muscles. By reflecting the iliac muscle sub-periosteally, the bony ilium was exposed. A retractor was used to expose the donor site. Two horizontal and five vertical cuts were made to harvest corticocancellous bone. The upper horizontal cut was placed midcrestal using a reciprocating saw. Four centimetres inferior, in the inner table, the other cut was made with a curved osteotome. These were connected by the vertical cuts using a reciprocating saw. After piece by piece removal of the corticocancellous bone blocks, additional cancellous bone was harvested with gouges and curettes [2]. After smoothing the bone and placement of Gelfoam (Upjohn, Kalamazoo, MI) in the bony cavity, the wound was closed in layers. Routinely, a suction drain was not placed.

All the operations were performed by the same experienced oral and maxillofacial surgeon at the UMCG. After harvesting the calvarial or iliac crest bone, maxillary augmentation surgery was performed according to the procedure by Raghoebar et al. [17].

Broad-spectrum antibiotics (amoxicillin/clavulanic acid, 625 mg t.i.d.) and non-steroidal anti-inflammatory drugs (ibuprofen, max. 600 mg t.i.d.) were provided for 1 week post-surgery. Patient instructions included a soft diet and chlorhexidine mouth rinse (1 min, two times daily) for 2 weeks. Two weeks after surgery, the dental prostheses were corrected and the patients were allowed to wear them again.

A 4-month healing time was considered to be sufficient for reliable placement of dental implants in regions grafted with anterior iliac crest and calvarial bone [18]. Therefore, it was chosen to place the implants in the maxilla 4 months after grafting irrespective of which bone was used for grafting. Next, after a 3-month osseointegration phase, all participants received their implant-supported maxillary overdentures. All participants were enrolled in a dental hygiene protocol.

### PROMs

#### OHRQoL assessment: OHIP-49NL

OHRQoL was assessed using the validated Dutch version of the Oral Health Impact Profile questionnaire (OHIP-49NL) [19–21]. This 49-item questionnaire assesses improvement or regression in a patient’s OHRQoL, enabling an analysis of any changes in OHRQoL over time. The questions are divided into seven domains describing different oral health impact problems: functional limitations, physical pain, psychological discomfort, physical disability, psychological disability, social disability and handicap. Patients have to complete five categories per question (graded 0–4) indicating how frequently a certain situation occurs (never, hardly ever, sometimes, fairly often or very often). A high OHIP-49NL score corresponds to a low OHRQoL. In this study, the OHIP scores were analysed according to an ordinal scale. The internal reliability, test/retest reliability and OHIP-49NL validity have been previously established [20, 22]. The Dutch version of the questionnaire, which has been evaluated for reliability and validity [19], was used for the current study.

#### Denture satisfaction questionnaire

Patient-reported denture satisfaction, including functional problem complaints in general, specific features related to facial and denture aesthetics and accidental lip, cheek and tongue biting, were assessed using a validated questionnaire [23]. The patients were asked to report the applicability of 40 denture-related complaints to their situation using a 4-point scale (0 = no complaints, 1 = few complaints, 2 = moderate complaints, 3 = severe complaints), with a lower score indicating a higher satisfaction.

#### Chewing ability questionnaire

Patients’ eating ability was assessed by a validated chewing ability questionnaire [24]. This questionnaire focuses on how patients experience eating soft, tough and hard foods and has three answer options: 0 = good, 1= moderate and 2 = bad. A lower score equals a better outcome as it indicates better chewing ability.

#### Direct post-operative pain

Each patient was asked to score the post-operative pain felt at the donor site during each of the first 30 days after harvesting surgery was performed. A 10-cm vertical visual analogue scale (VAS) score was used, with the bottom anchor representing ‘no pain’ and the top anchor as ‘worst pain imaginable’. Assessments took place at 12 o’clock each day. By measuring the distance (millimetres) on the 10-cm line between the ‘no pain’ anchor and the patient’s mark, the score is determined on a range from 0 to 100. For interpretation of the scores, the following cut points on the pain VAS were used: no pain (0–4 mm), mild pain (5–44 mm), moderate pain (45–74 mm) and severe pain (75–100 mm).

#### Patient satisfaction with the procedure and outcomes

A three-item list questioned several aspects of the patient’s experience with the procedure. The patient’s satisfaction with the end result was assessed using a 10-cm VAS scale with the bottom anchor representing ‘very unsatisfied’ and the upper anchor ‘very satisfied’. The other two items questioned (yes/no) whether the patient would recommend the procedure to other patients with the same problem and whether the patient would be willing to undergo the same operation if needed. Furthermore, satisfaction with the outcomes was assessed regarding the scar aesthetics at the donor site (yes/no) and whether the altered donor site contour was bothersome (yes/no).

#### Long-term sequela

Twelve months after the implant-based prostheses’ were placed, the patients were seen for the final follow-up. They were asked to rate the current pain at the donor site (VAS score). In addition, the patients were questioned regarding difficulties with wearing clothes (wearing a hat/cap, a belt or a pair of trousers) and difficulties with functional mobility (complaints during walking, climbing stairs or cycling). Patients were asked whether they had perceived such difficulties during the 7 days prior to the follow-up meeting and whether these problems had been present before surgery. If the latter was positive, the results were excluded from the evaluation. The items were formulated as two-choice questions (yes/no).

### Statistical analysis

The data were collected by one observer (ABE). Data management and analysis were performed using SPSS 23.0. Data were tested for normal distribution with a Shiparo-Wilk test and checked visually using a histogram with a distribution curve. If required, the outcomes of a non-normally distributed variable were transformed into a normal distribution using a Log10 transformation. The Student *t* test, the Mann-Whitney *U* test and the Pearson *χ*^2^ test compared the outcomes of the parametric variables, nonparametric variables and the categorical gender variable between groups, respectively. Concerning the outcome data, the Pearson *χ*^2^ test compared dichotomous variables. For the post-operative pain diary, a mixed ANCOVA was performed. Medians instead of means were calculated for non-normally distributed continuous variables such as the general satisfaction (VAS score) and questionnaire scores. A significance level of 0.05 was chosen for all tests.

## Results

All consecutive eligible patients that were referred to our department between November 2014 and March 2016, and met the inclusion criteria, were willing to join the study. The augmentation surgery resulted in sufficient bone volume for implant placement at the prosthodontically preferred sites in all cases. No peri-operative complications occurred and no additional interventions, such as drain placement at the donor site, were needed. A total of 44 implants was placed in each group. In each group, one patient lost an implant because of mobility during the osseointegration phase, resulting in a 1-year implant survival rate of 97.7%. The clinical characteristics of both groups are listed in Table 1.

**Table 1:** Characteristics of the study group

	Total group	Calvarium group	Anterior iliac crest group	Comparing groups	
	*n* = 20	*n* = 10	*n* = 10	Test statistic	p-value*
Sex				Pearson-χ^2^-test	
Male	9	5	4	0.202	1.000
Female	11	5	6		
Number of implants placed					
Participants with 4 implants	10	8	8		
Participants with 6 implants	10	2	2		
Number of implants lost	2	1	1		
	*Median (IQR)*	*Median (IQR)*	*Median (IQR)*	Mann-Whitney U	
Age at implant placement (years)	65.4 (56.4;71.1)	68.4 (54.6;72.7)	63.5 (56.5;69.3)	41.000	.529
Time between augmentation and implant placement (days)	133 (126;145)	140 (131;152)	126 (119;133)	17.500	.011

Results are presented as the number or the median (interquartile ranges: IQR)

*Exact sig. (2-sided)

### OHIP-49NL, denture satisfaction and chewing ability

#### OHIP-49NL

The OHIP-49NL sum scores and scores on all seven domains improved between baseline and 12 months post denture placement (Wilcoxon signed-rank test, *p* = 0.001–0.003, Table 2). The functional limitation and physical disability domains showed the largest improvements, whereas psychological discomfort, social disability and handicap improved the least. The OHIP-49NL scores showed no significant differences in improvement scores between the groups (Mann-Whitney *U* test, *u* = 34.00–49.50, *p* = 0.23–0.98, Table 3).

**Table 2:** OHIP-49NL. Denture satisfaction and chewing ability scores: pre- and post-treatment results for all participants

Questionnaire n = 20		Pre-treatment measurement	Post-treatment measurement	Score change	
	Max. Pos.^a^	Median (IQR)	Median (IQR)	Wilcoxon signed rank-test (Z-value	*p*-value
OHIP-49NL					
Functional limitation	36	17.44 (15.25; 24.75)	3.69 (1.34; 7.00)	-3.846	.000
Physical Pain	36	14.50 (10.50; 10.00)	3.00 (0.20; 13.75)	-2.829	.000
Psychological discomfort	20	11.00 (8.40; 16.00)	0.50 (0.00; 7.00)	-3.829	.000
Physical disability	36	16.50 (9.20; 24.25)	2.00 (0.00; 8.50)	-3.785	.000
Psychological disability	24	8.50 (1.20; 15.50)	0.00 (0.00; 3.00)	-3.590	.000
Social disability	20	3.50 (0.00; 9.50)	0.00 (0.00; 4.00)	-2.991	.001
Handicap	24	4.00 (1.00; 11.25)	0.00 (0.00; 0.75)	-3.526	.000
Summary scores	196	78.80 (51.75; 125.12)	16.00(3.25; 40.00)	-3.883	.000
Denture satisfaction	216	94.53 (84.25; 121.00)	61.00 (56.38; 74.30)	-3.92	.000
Chewing ability	27	16.00 (13.00; 18.00)	11.00(9.00; 13.00)	-3.340	.000

Results are presented as the median (interquartile ranges: IQR)

^a^Maximum score possible on test/domain

**Table 3:** OHIP-49 scores, denture satisfaction and chewing ability: group score changes following treatment

Questionnaire *n* = 20	Calvarium group	Anterior iliac crest group	Comparing groups	
	Median (IQR)	Median (IQR)	Mann-Whitney U	*p*-*p*-value*^1^
OHIP-49				
Functional limitation	9.69 (5.50; 15.75)	13.44(9.66; 20.41)	34.00	.24
Physical pain	5.19 (-2.00; 15.85)	12.00(1.50; 22.75)	39.00	.42
Psychological discomfort	9.00 (1.75; 12.00)	11.00(5.50; 13.25)	36.00	.30
Physical disability	12.50(0.75;17.50)	10.50(9.00;19.50)	43.50	.64
Psychological disability	5.00(1.50;11.25)	5.00(1.00;12.25)	49.50	.98
Social disability	1.50(-0.25;4.00)	2.00(0.00;8.50)	39.50	.44
Handicap	1.50(0.00;5.25)	4.00(1.00;9.75)	34.00	.23
Summary scores	51.39(14.67;85.79)	61.80(26.08;92.14)	39.00	.44
Denture satisfaction	12.34(4.37; 54.80)	39.02(27.95; 70.40)	27.00	.09
Chewing ability	5.00(-0.75;7.28)	4.50(2.75; 7.50)	43.00	.61

Results are presented as median (interquartile ranges: IQR).

*^1^ Exact sig. (2-sided)

#### Denture satisfaction

The scores improved significantly after treatment (median score 61.00 (IQR 56.38, 74.30) (Wilcoxon signed-rank test, *p* = 0.001, Table 2) and were similar in both groups (Mann-Whitney *U* test, *u* = 27.00, *p* = 0.09, Table 3).

#### Chewing ability

Chewing ability improved from 16.00 (IQR 13.00, 18.00) at baseline to 11.00 (IQR 9.00, 13.00) 12 months after overdenture placement (Wilcoxon signed-rank test, *p* < 0.0001, Table 2), and the group outcomes were also similar (Mann-Whitney *U* test, respectively, *u* = 27.00, *p* = 0.09 and *u* = 43.00 *p* = 0.61, Table 3).

### Direct post-operative pain

**Fig. 2:**
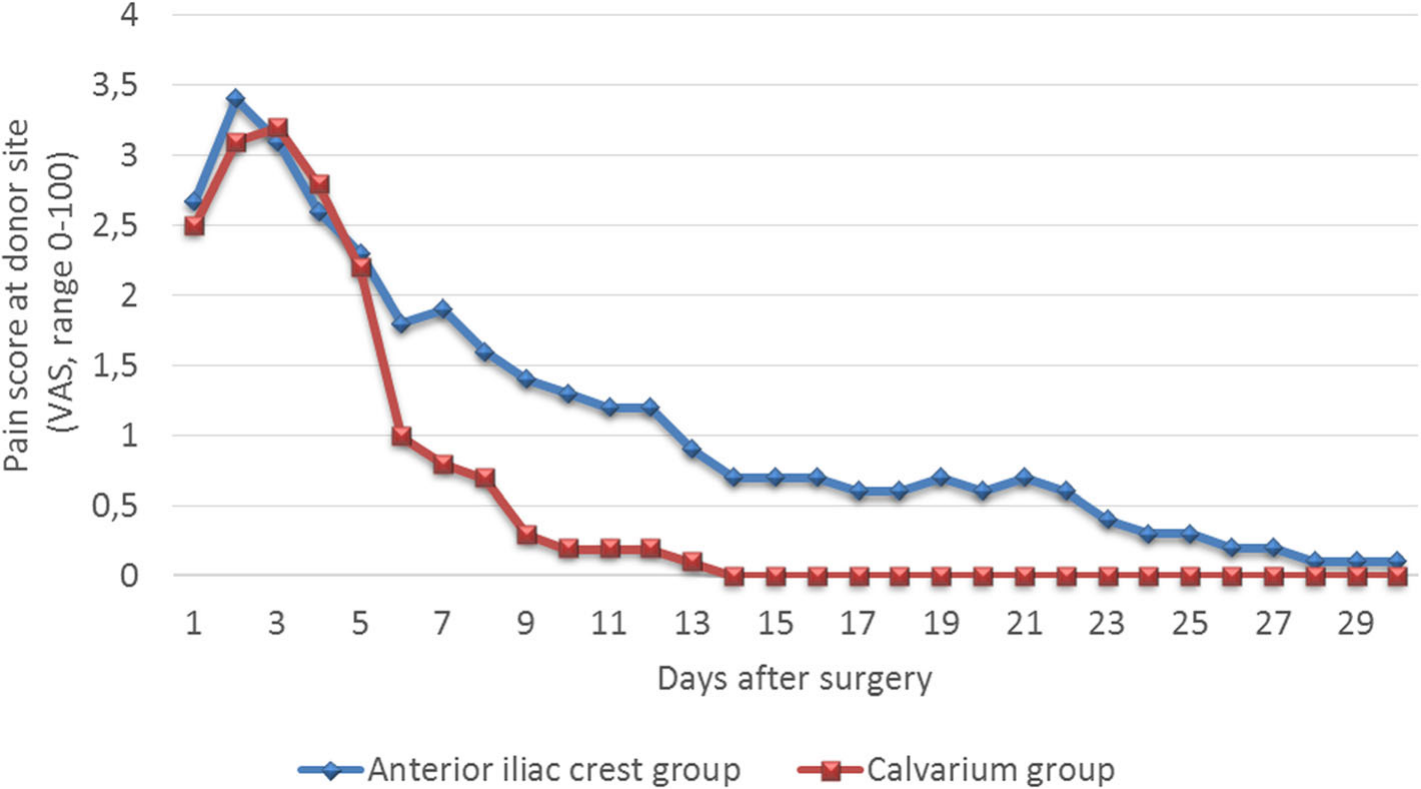
Significant difference between groups in mean post-operative pain at donor site. During the first 30 days following maxillary reconstruction with either calvarial (*n* = 10) or anterior iliac crest (*n* = 10) bone grafts, participants scored the pain felt at donor site using a 100-mm VAS scale (‘0’ represents ‘no pain’ and ‘100’ represents ‘worst pain ever’). The mean VAS scores for pain ranged from 32.5 ± 17.1 (day 2) to 3.5 ± 15.8 mm (day 14). A linear mixed model determined a significant difference between treatment groups with an estimated effect of 0.09 (standard error = 0.015) for the anterior iliac crest group (*G* = 31.3, *p* = 0.00), meaning the pain scores of anterior iliac crest group are higher than the calvarium group scores (*F* = 31.30, *p* < 0.00)

The mean VAS scores for pain ranged from 32.5 ± 17.1 on day 2, which can be interpreted as mild pain, to 3.5 ± 15.8 mm on day 14, interpreted as no pain (Fig. 2). After a Log10 transformation of the data to correct for skewness, a linear mixed model was run to determine and to compare the course of pain scores between the treatment groups. There was a significant difference between treatment groups with an estimated effect of 0.09 (standard error = 0.015) for the anterior iliac crest group (*G* = 31.3, *p* = 0.00), meaning the pain scores of anterior iliac crest group are higher than the calvarium group scores (*F* = 31.30, *p* < 0.00).

To determine the effect of time and covariates such as age, gender and BMI on the VAS scores, a repeated measures ANCOVA was run. Mauchly’s test of sphericity indicated that the assumption of sphericity had been violated (*X*^2^ = 0.000, *p* < 0.0005), and therefore, a Greenhouse-Geisser correction (*ɛ* = 0.11) was used. There was a significant effect of time on VAS scores, *F*(3.1;55.3) = 32.6, *p* < 0.0005. (Fig. 2). Furthermore, an interaction was found between BMI and VAS scores of the anterior iliac crest group (Greenhouse-Geisser, *ɛ* = 0.14, *F*(3.3;26.4) = 2.9, *p* = 0.04), but not for the calvarium group (Greenhouse-Geisser, *ɛ* = 0.084, *F*(2.4;19.5) = 0.1, *p* = 0.93).

### Patients’ satisfaction

The results on general patient satisfaction are listed in Table 4. All the participants (*n* = 20) confirmed that they would undergo the same procedure again if needed and that they would recommend the procedure to others. The overall level of satisfaction with the end result was high with a median of 93 (IQR 86, 99) on a 100-mm VAS scale (*n* = 20).

**Table 4:** General satisfaction: results for all participants and per group

	All participants *n* = 19*^1^		Calvarium group *n* = 9*^1^		Iliac crest group *n* = 10		Comparing groups	
	Median (IQR)		Median (IQR)		Median (IQR)		Mann-Whitney U	*p*-value*^1,2^
How satisfied are you concerning the end result? (VAS-score in mm)	93 (86;99)		87(74;100)		95(90;95)		34.500	.247
	*Yes*	*No*	*Yes*	*No*	*Yes*	*No*		
Would you recommend the procedure to other patients with the same problem?	20	0	10	0	10	0		
Would you be willing to undergo the same operation when needed?	20	0	10	0	10	0		

Results are presented as the number or the median (interquartile ranges: IQR)

*^1^ After excluding one outlier from the calvarium group who reported a VAS-score of 4mm

*^2^ Exact sig. (2-sided)

On separating the results according to treatment group, the median VAS score of the calvarium group was 87 mm (IQR 74, 100) and of the anterior iliac crest group, 95 mm (IQR 90, 100) (Mann-Whitney *U* test, *U* = 34.5, *p* = 0.247). The VAS scores on satisfaction with the end result contained one outlier (VAS score 4 mm) in the calvarium group. The final appearance of the prosthetic device did not match this patient’s expectations. The complaint was directed at the prosthetic technique and not at the surgical procedure. On excluding this case from the analysis, the remaining scores provided a median score of 93 mm (IQR 86, 99) for the entire study group (*n* = 19) and 89 mm (IQR 81, 100) for the calvarium group (*n* = 9). There was no significant difference either when the median VAS scores were compared without the outlier (Mann-Whitney *U* test, *U* = 34.00, *p* = 0.400).

#### Donor site appearance

Regarding changes at the donor site, one patient from each treatment group noticed an alteration in the contour. Two patients from the anterior iliac crest regarded the scar aesthetics as being acceptable instead of satisfactory (Pearson *χ*^2^ test, 2.222, *p* value = 0.474).

### Long-term sequelae

#### Pain

The median VAS scores for current donor site pain at the calvarium and anterior iliac crest site were 1 mm (IQR 0, 1 mm) and 2 mm (IQR 1, 3), respectively. (Mann-Whitney *U* test, *U* = 30.500, *p =* 1.000 for the current pain at donor site) (Table 5).

**Table 5:** Patient reported outcomes of bone graft harvesting surgery per group

	Calvarium group *n* = 10		Iliac crest group *n* = 10		Comparing groups	
	Median (IQR)		Median (IQR)		Mann-Whitney U	*p*-value*^1^
Donor site pain (VAS-scores)						
How would you rank the current pain felt at the donor site?	1(0;1)		2(1;3)		30.500	1.000
Donor site related complaints in daily functioning	Yes	No	Yes	No	Pearson-χ^2^ test	
During the past week, did you perceive any of the following						
Headache	2	8	2	8	.000	1.000
Difficulties with wearing cloths^*2^:	0	10	1	9	1.053	.305
Difficulties with functional mobility ^*3*4^	0	10	1	9	1.053	.305
Are you satisfied with the scar aesthetics at the donor site?	10	0	8	2	2.222	.474
Do you consider the altered contour of the donor site bothersome?	1	9	1	9	0.000	1.000

Results are presented as the number or the median (interquartile ranges: IQR)

*^1^ Exact sig. (2-sided)

^*2^Difficulties with wearing daily cloths such as a hat, cap, belt or pair of trousers

^*3^Difficulties with getting around in daily living, such as with walking, climbing the stairs or cycling

^*4^Statistical test performed exclusive of one case with pre-surgical difficulties on functional mobility

#### Daily functioning complaints

None of the participants in the calvarium group reported difficulties with wearing clothes or functional mobility (Table 5). One participant in the anterior iliac crest group reported difficulties with wearing clothes. Furthermore, two participants from the anterior iliac crest group noted they had problems with functional mobility. One of these two patients reported pre-surgical problems with walking as well. It was unclear whether the complaints were stable or had worsened or improved. The differences between the groups were not statistically significant (Pearson *χ*^2^ test, *p* values 0.31–1.00, Table 2).

## Discussion

PROMs are a core aspect in treatment programme evaluations [25]. Therefore, patients’ appreciation of extraoral bone graft harvesting, used for pre-implant augmentation of the edentulous maxilla, was assessed. The bone graft harvesting surgery itself and the complete procedure enabled by the bone grafting showed a high patient-reported satisfaction with the course and its results. The PROMs imply a successful treatment, and apart for the higher post-operative pain scores following harvesting anterior iliac crest bone, the outcomes are similar for calvarial and iliac crest bone harvesting.

This study’s results are in accordance with previous findings in literature on OHRQoL, denture satisfaction and chewing ability, procedure-related satisfaction and long-term donor site-related outcomes [9]. The prospective, controlled design of this study enables confirmation of the suggested similarities between the procedures from a patient’s point of view. For clinical decision-making, the interaction between direct post-operative pain and BMI can be taken into account. Furthermore, the minor differences in satisfaction with the outcomes at donor site and problems with physical mobility should be considered as well.

Another previously described phenomenon was found: the surgery comes along with moderate direct post-operative pain and with high levels of satisfaction [14]. High pain levels following extraoral bone harvesting [8], especially when it comes to the anterior iliac crest [3, 26], are frequently mentioned as a major disadvantage from a patient’s perspective, and the coexistence with high satisfaction with the procedure is a frequent subject of debate [27]. This discussion might result from the way the patient satisfaction construct is interpreted. A complete model of this construct can explain this coexistence. Patient satisfaction covers all aspects of care quality, that is, appropriate access to health services, provision of health information, relationship between patient and health care staff, participation in making choices regarding health treatment, satisfaction with the treatment provided, effectiveness of treatment including the extent to which the treatment meets the patient’s expectations of care, and general satisfaction [28]. Thus, a patient’s satisfaction with treatment is not dictated exclusively by physical parameters [28], and therefore, it can be high despite moderate post-operative pain.

This study assessed satisfaction at the final follow-up to assure the patients’ appreciation would entail each step in the treatment programme. However, the course that patients’ satisfaction makes was not registered. Furthermore, not all dimensions of patients’ satisfaction were assessed as this study focussed on the patients’ appreciation of the technical procedure. Future research on these two points can help in improving the treatment programme. Furthermore, it is worth noting that in the current study, the positive PROMs are measured in complete absence of post-operative complications in both groups. As the complication rate for both treatments is low, we consider the results of our study as being representative.

To conclude, prosthetic rehabilitation programmes, encompassing maxillary augmentation with extraoral bone grafts from either the calvarium or anterior iliac crest, are reliable pre-implant surgery procedures for extremely resorbed maxilla cases, as they are associated with high patient satisfaction in terms of both treatment procedure and end results. As patient satisfaction is determined by the patient’s expectations and provision of information, an explanation of the procedure and the course of post-operative complaints deserves special attention in clinical practice.

## Data Availability

The datasets used and/or analysed during the current study are available from the corresponding author on reasonable request.

## Abbreviations

CBCT: Cone-beam computed tomography
IQR: Interquartile range
OHIP-49NL: Oral Health Impact Profile, 49-item version in Dutch
OHRQoL: Oral health-related quality of life
PROM: Patient-reported outcome measures
UMCG: University Medical Centre Groningen
VAS: Visual analogue scale
